# Employing crisis postcards with case management in Kaohsiung, Taiwan: 6-month outcomes of a randomised controlled trial for suicide attempters

**DOI:** 10.1186/1471-244X-13-191

**Published:** 2013-07-17

**Authors:** Wei-Jen Chen, Chi-Kung Ho, Shi-Sen Shyu, Cheng-Chung Chen, Guei-Ging Lin, Li-Shiu Chou, Yun-Ju Fang, Pin-Yang Yeh, Tieh-Chi Chung, Frank Huang-Chih Chou

**Affiliations:** 1 Kaohsiung City Municipal Kai-Syuan Psychiatric Hospital, 130, Kai-Syuan 2nd Road, Ling-Ya District, Kaohsiung 802, Taiwan; 2 Graduate Institute of Counselling Psychology and Rehabilitation Counselling, National Kaohsiung Normal University, Kaohsiung, Taiwan; 3 Kaohsiung City Government Bureau of Health, Kaohsiung, Taiwan; 4 Institute of Health Care, Meiho University, Neipu, Pingtung, Taiwan

**Keywords:** Suicide re-attempts, Case management, Crisis postcard

## Abstract

**Background:**

Suicide attempts constitute a serious clinical problem and have important implications for healthcare resources. The aim of the present study was to evaluate the effectiveness of case management using crisis postcards over a 6-month follow-up period.

**Method:**

A randomised controlled trial was conducted in Kaohsiung, Taiwan. Prevention of further suicide attempts was compared between two groups with and without the postcard intervention. The intervention group consisted of 373 participants (139 males, 234 females; age: 39.8 ± 14.0 yrs.). The control group consisted of 388 participants (113 males, 275 females; age: 40.0 ± 16.0 yrs.). A survival analysis was used to test the effectiveness of the crisis postcard intervention for the prevention of suicide reattempts. Per-protocol and intention-to-treat analyses were conducted.

**Results:**

The intention-to-treat analysis indicated that the crisis postcard had no effect (hazard ratio = 0.84; 95% CI = 0.56 – 1.29), whereas the per-protocol analysis showed a strong benefit for the crisis postcard (hazard ratio = 0.39; 95% CI = 0.21 – 0.72).

**Conclusion:**

Although the results of the present study indicated that the postcard intervention did not reduce subsequent suicide behaviour, our study provides an alteration to the postcard intervention. Further studies need to be conducted to clarify whether this type of intervention can reduce subsequent suicidal behaviour, with a particular focus on reducing the rate of loss to follow-up.

## Background

Suicide is a global challenge and a major public health problem worldwide. Suicide accounts for almost 1 million deaths annually, and an estimated 10 million suicide attempts occur every year [1]. Approximately 32% of all deaths due to suicide occur in the Western Pacific region [2]. This disproportionately high rate of suicide is geographically constrained to 37 countries and an estimated 29% of the world’s population. The suicide rate in this region is approximately 19.3 per 100,000 individuals [3]. According to the 2011 report by the Department of Health, Executive Yuan in Taiwan, suicide was one of the ten leading causes of death in the nation between 1998 and 2010. The suicide rate was 16.8 per 100,000 individuals in 2010. In Kaohsiung City, Taiwan’s second largest metropolitan city, suicide has become a major public health issue, a fact that is reflected in a relatively high suicide rate, which reached 18.4 deaths for every 100,000 people in 2010.

The repetition rate of self-harm reported in a review of findings from Western countries is 15-16% at 1 year with a slow increase to 20-25% over the following few years [4]. In Taiwan, the cumulative risk of non-fatal repetitions of self-harm is 5.7% within the first year and 9.5% over 4 years [5]. Suicidal behaviours are multifactorial phenomena associated with a range of negative outcomes, most notably, the risk of another suicide attempt, a completed suicide, and other forms of premature mortality [6-8]. However, Hawton and colleagues have noted that there is insufficient evidence on which to make firm recommendations about the most effective forms of treatment for people who have recently deliberately harmed themselves [9].

Cognitive behavioural therapy is a pragmatic, action-oriented intervention for major mental disorders, and its methods are modified for use in many other conditions [10]. One particularly useful way to encourage clients to use behavioural skills learned in therapy sessions is to develop a coping card [11]. The key elements of a coping strategy are recorded on a small card that the patient carries with them at all times. These coping cards might contain, for instance, an anti-suicide plan that details what to do if suicidal thoughts return. Coping strategies that are generated and rehearsed in intervention sessions are then utilised in real life with coping cards. The “postcard” intervention is first proposed by Motto [12]. Research shows that contacting at-risk people via letter or postcard can reduce suicide risk [13]. The postcard intervention can reduce repeated suicide attempts after discharge for deliberate self-poisoning [14,15]. A “crisis card” contains the details of a patient’s treatment plan in anticipation of a later occasion when the patient might be too ill to remember their treatment plan [16].

The gold standard used to evaluate the efficacy of an intervention is a randomised controlled trial, but few studies have successfully tested suicide interventions using a randomised controlled trial design [13]. Due to ethical considerations, it is difficult to deny any intervention or treatment to individuals in this high-risk population [17]. Therefore, ethical constraints prevented us from randomly assigning subjects to a control group that was denied any treatment and an intervention group that used a crisis postcard intervention to test the postcard’s effectiveness. Of the existing randomised controlled trials, it is noteworthy that most used an add-on intervention versus a standard intervention trial design [18,19].

Based on these constraints, we added a crisis postcard intervention to case management services to treat individuals who had repeatedly attempted suicide. The aim of the present study was to evaluate whether the use of crisis postcards in addition to case management is more effective than case management alone in preventing suicide reattempts over a six-month follow-up period.

## Methods

### Location

Kaohsiung City is located on the southwest coast of Taiwan and is the second largest city in Taiwan. It features a highly developed manufacturing industry and is home to the largest commercial harbour in Taiwan. Kaohsiung City plays a pivotal role in commercial industry and shipping in Taiwan and is an important port between the Indian Ocean and northeast Asia.

### Subjects

When individuals who had attempted suicide within the previous month were found by suicide prevention gatekeepers in medical or non-medical organisations in Kaohsiung, gatekeepers filled in the relevant information about the suicide attempters on the national suicide prevention reporting sheet and faxed them to the suicide prevention centre in Kaohsiung according to our national suicide prevention policy. The eligible participants in our study were recruited from July 2011 to December 2011. Referred cases included hospitalised patients, patients identified by the Bureau of Police and the Bureau of Fire, and patients identified and referred by suicide prevention gatekeepers.

### Randomisation and study profile

After receiving the participants’ documented informed consent, they were randomly assigned to either the intervention group or the control group according to the last digit of their National Identification (ID) Card. In Taiwan, every citizen, when he/she is born, is randomly assigned an ID number by the National Department of Internal Affairs, regardless of sex, race, skin colour and place of birth. A valid National Identification number consists of one English letter and is followed by nine digits. Because the last digit of the ID (from 0 to 9) has the same probability of being chosen, it was used for randomisation during the sampling process in this study. Participants with an ID card ending in an odd number were assigned to the intervention group, and those with an ID ending in an even number were assigned to the control group.

**Figure 1:**
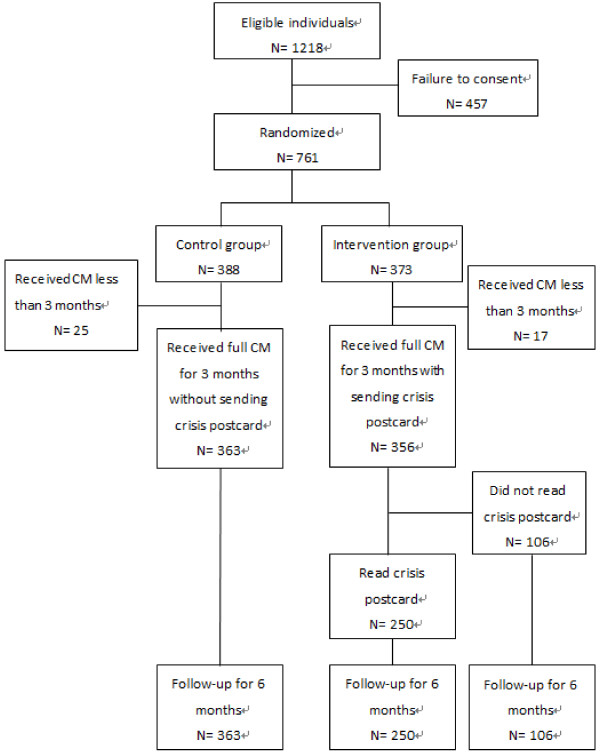
Flow diagram of the study (CM: case management).

The process of enrolment is illustrated in Figure 1, and the study profile was reviewed and approved by the Institutional Review Board of Kai-Syuan Psychiatric Hospital.

### Procedures for case management and the crisis postcard intervention

The case managers of the suicide prevention centre in Kaohsiung are typically psychiatric nurses, but our team also includes psychologists and social workers. After receiving the subjects’ information from the national suicide prevention reporting sheets, we contacted the suicide attempters via telephone or a home visit within one week and provided case management services at least six times in the following three months. The case management services included psychological support, providing proper coping strategies, follow-ups to increase adherence to the referrals provided for psychiatric treatment, and individualised casework, including the coordination of social resources and brief crisis intervention if needed [20].

**Figure 2:**
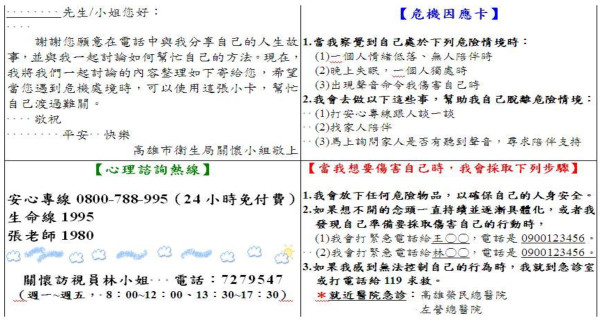
Examples of crisis postcards (in Chinese).

Case managers at the suicide prevention centre gathered the subjects’ individualised information related to the crisis and discussed it with the subjects during the case management services. Afterwards, the case managers listed the necessary coping strategies and appropriate resources on the crisis postcard. There were two major components included on our crisis postcards: [1] individual coping strategies and [2] resources that can help a suicide attempter to overcome obstacles during a crisis (Figure 2). The crisis card was small enough to fit into a wallet or pocket so that the suicide attempter could carry it at all times. A single crisis postcard was sent to a participant in a sealed envelope after 3 months of full case management services in the intervention group. Several key elements of our crisis postcard differed from the green card study and other various postcard studies in style, timing and content [14,15,21,22]. The intervention of our study was individually tailored to the suicide attempter based on the case management assessment and services. One month after sending the crisis postcards, we asked the participants in the intervention group whether they had read their crisis postcards.

### Variables

Variables on the national suicide prevention reporting sheet consisted of categorical variables and continuous variables. The categorical variables were sex, referral source (i.e., if subjects were referred from a medical organisation), employment (i.e., employed or not), living conditions (i.e., if they lived alone) and suicide method. High lethality suicide methods included hanging, charcoal burning or jumping from high altitudes. The continuous variable was age. The history of suicide attempts was acquired from the record of the National Suicide Prevention Reporting System in our study.

### Statistical analyses

The primary outcome measure was the occurrence of a suicide reattempt during a 6 month follow-up period. The primary objective of this study was to examine whether the time period until recurrent suicidal behaviour of participants in the intervention group was significantly different from that of the control group. The primary endpoint was based on a survival analysis and determined using the Cox proportional hazard model to test the effectiveness of the crisis postcard intervention on the prevention of suicide reattempts. The length of follow-up for each subject was represented by the number of days between the baseline evaluation and the date of either a suicide reattempt or the end of the follow-up period, depending on which came first. The results are reported as hazard ratios, 95% confidence intervals (CIs) and p-values from the Cox proportional hazard regression model. Statistical significance was considered to be p < .05. Survival and Cox regression analyses were conducted using the Statistical Package for the Social Sciences (version 17.0). Per-protocol (subjects receiving full case management alone for 3 months vs subjects receiving full case management for 3 months who read the crisis postcard) and intention-to-treat (ITT) analyses (control group vs intervention group) were conducted in our study. The differences between the demographic characteristics of the present study were tested using the *χ*^2^ test and the independent samples *T* test.

## Results

We assessed 1,218 participants for eligibility, of whom 457 failed to give consent (Figure 1), leaving 761 participants – 388 in the control group and 373 in the intervention group. Full case management services were received by 363 participants in the control group and 356 participants in the intervention group. Within the intervention group, 250 subjects read the crisis postcards. Table 1 shows the demographic characteristics of the participants.

**Table 1:** Demographic characteristics of the study sample

	**ITT analysis**			**Per-protocol analysis**				
	**Control group, N = 388**	**Intervention group, N = 373**	**p**^**a**^	**Control group who received full CM alone, N = 363**	**Intervention group who received full CM, N = 356**			**P**^**b**^
					**Read crisis postcard, N = 250**	**Did not read crisis postcard, N = 106**	**P**^**c**^	
**Categorical variables, N (%)**								
Female	275 (70.9)	243 (65.1)	.09	235 (64.7)	181 (72.0)	70 (66.0)	.26	.06
Referred from medical organisation	342 (88.1)	317 (85.0)	.2	311 (85.7)	220 (87.6)	96 (90.6)	.42	.49
Employment	154 (39.7)	126 (33.8)	.09	124 (34.2)	104 (41.2)	37 (34.9)	.27	.08
High lethality suicide method	44 (11.3)	37 (9.9)	.52	34 (9.4)	29 (11.6)	13 (12.3)	.85	.37
History of previous suicide attempt	149 (38.4)	130 (34.9)	.31	129 (35.5)	86 (34.4)	53 (50)	.01	.77
Live alone	63 (16.2)	66 (17.7)	.59	62 (17.1)	30 (12.0)	19 (17.9)	.03	.08
**Continuous variables, mean ± SD**								
Age (yrs)	40.0 ± 16.0	39.8 ± 14.0	.06	40.1 ± 16.2	39.0 ± 14.0	40.6 ± 12.9	.36	.06

P^a^: P value of comparison between subjects in the control group and those in the intervention group.

P^b^: P value of comparison between subjects in the intervention group who received full CM and those in the control group who received full CM.

P^c^: P value of comparison between subjects in the intervention group who received full CM and read the crisis postcard and those in the intervention group who received full CM but did not read the crisis postcard.

### Suicide reattempts

The mean time to suicide reattempt of subjects in the control group of subjects in the intervention group of subjects in the control group who received full case management alone, and of subjects in the intervention group who received full case management and read the crisis postcard were 166.8 days, 170.7 days, 166.9 days and 177.3 days, respectively.

The Cox proportional hazard model was conducted to test the effectiveness of the crisis postcard intervention on the prevention of suicide reattempts. The ITT analysis showed no effect for the crisis postcard (hazard ratio = 0.84; 95% CI = 0.56 – 1.29), but the per-protocol analysis showed a strong benefit for the crisis postcard (hazard ratio = 0.39; 95% CI = 0.21 – 0.72).

**Figure 3:**
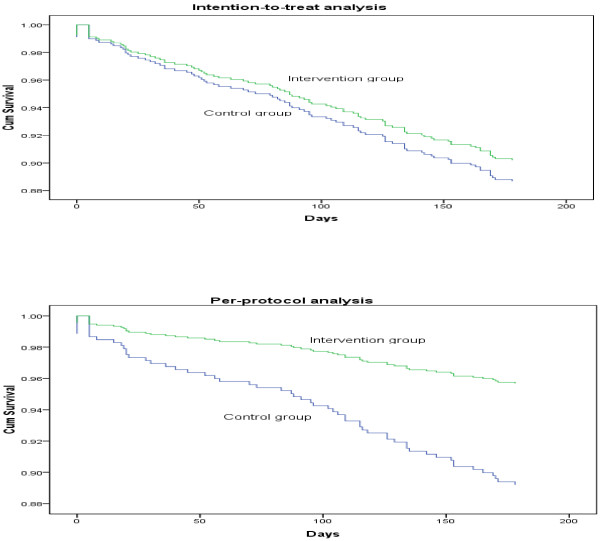
Survival curves comparing the time to suicide reattempt between the control group and the intervention group by intention-to-treat analysis (HR = 0.84, 95% CI = 0.56 – 1.29) and per-protocol analysis (HR = 0.39, 95% CI = 0.21 – 0.72).

A survival analysis (Figure 3) was used to create survival curves comparing the time to suicide reattempt between the control group and the intervention group in the intention-to-treat and per-protocol analyses.

## Discussion

Although the per-protocol analysis showed a strong benefit for the crisis postcard, the primary finding of the present study was that the crisis postcard intervention did not significantly reduce subsequent suicidal behaviour in the intention-to-treat analysis. Excluding non-adherent suicide attempters might introduce bias because suicide attempters who adhered to treatments tend to have better outcome regardless of whether the treatment was effective. Although there were no significant differences between subjects receiving full case management services who read the crisis postcards in the intervention group and those receiving full case management alone in the control group, precautious measures should be taken when considering the per-protocol analysis. The 106 subjects excluded from the intervention group had a significantly higher proportion of patients with a previous history of suicide attempt. This might contribute to a pre-existing difference and produce potential biases in a per-protocol analysis. In addition, excluding those who did not read the postcards could bias the apparent effectiveness. Suicide attempters who would like to read the crisis postcards might have better outcomes than those who would not read the crisis postcards, regardless of treatment. Because the per-protocol analysis removed the balance of randomisation, the results were more similar to those of an observational study than a randomised trial and should be interpreted with caution.

When analysing data from randomised clinical trials, intention-to-treat analysis provides the highest level of evidence. However, the intention-to-treat analysis almost certainly underestimated the effectiveness of the crisis postcard. Because the intervention group was “contaminated” with participants who received the crisis postcard but did not read it, the groups appeared more similar, and it was harder to detect a difference between them (if one existed). Thus, the intention-to-treat analysis might have had only a slim chance of detecting a true difference between the groups.

The results of the present study are similar to those of a previous study conducted by Beautrais and colleagues, who reported that a postcard intervention did not significantly reduce self-harm reoccurrences [22]. However, the results of this study are in contrast to those of previous randomised controlled trials [14,15]. Carter and colleagues indicated that a postcard intervention reduced subsequent suicidal behaviour. There are four possible reasons that may account for this discrepancy. The first might be that the intervention in Carter’s study consisted of a series of the eight fixed-format postcards sent by mail during the 12 months following a participant’s index presentation for a suicide attempt or self-harm. However, the intervention in our study comprised a single individualised crisis postcard that included individual coping strategies and resources. The postcard was sent to the participants in a sealed envelope by mail after 3 months of case management services. The difference in the format and number of the postcards might contribute to this discrepancy. As a second possibility accounting for this discrepancy, the postcard intervention may have a different effect on various ‘suicidal’ behaviours. Participants in Carter’s study were restricted to those presenting with self-poisoning, whereas the sample used in our study included those presenting with any method of suicide attempt. Third, a higher proportion of the participants in our study had a history of a previous suicide attempt compared to the participants in Carter’s study. Last, the effectiveness of the postcard intervention relies on the level of social support already available and the overall treatment setting. The differences in the healthcare models may have resulted in different effects.

A limitation of this study was the relatively high rate of loss to follow-up in the intervention group, which was problematic when analysing the results. The elimination of patients lost to follow-up from the per-protocol analysis overestimated the effect of the crisis postcard, whereas the estimation of the treatment effect was generally conservative because of dilution due to non-compliance in the intention-to-treat analysis.

## Conclusion

The individualised crisis postcard in the current study altered the physical appearance and the wording of the card for each patient. Although the present study indicated that the postcard intervention did not reduce subsequent suicide behaviour, our study provided an alteration to the postcard intervention. Further studies are needed to clarify whether this type of intervention can reduce subsequent suicidal behaviour, with particular emphasis on reducing the rate of loss to follow-up.

## Competing interests

The authors declare that they have no competing interests.

## Authors’ contributions

WJC: The first author, who collects, analyses data, writes and reedits this manuscript. CKH: The second author, who helps first author to collect data and give suggestions and comments on the manuscript. SSS: The third author who helps first author to collect data and reedit the manuscript. CCC is equally shared with corresponding author with FHC, GGL, LSC, YJF, PYY, TCC who helps to collect data, participant in this study and give some suggestions on this manuscript FHC is the corresponding author who analyses raw data, writes partial paper and instructs the authors to write this manuscript. All authors read and approved the final manuscript.

## Pre-publication history

The pre-publication history for this paper can be accessed here:

http://www.biomedcentral.com/1471-244X/13/191/prepub
